# Neutrophils as Possible Therapeutic Targets in Severe Influenza Pneumonia

**DOI:** 10.16966/2470-3176.115

**Published:** 2016-05-31

**Authors:** Teluguakula Narasaraju, Ashar Harshini

**Affiliations:** Center for Veterinary Health Sciences, Oklahoma State University, Oklahoma, USA

The threat of pandemic and highly pathogenic influenza viruses currently represents a top global public health problem. Majority of deaths in patients with severe influenza infections are due to complications resulting from impaired gas-exchange, pulmonary edema and alveolar-capillary damage, which are characteristics of acute respiratory distress syndrome (ARDS) [1,2]. Although vaccination is the ultimate choice for preventing outbreaks and further pandemic spread of the virus, due to emergence of novel strains and the mutative ability of these viruses, effective vaccine is quite challenging. Treatment of infection using antiviral agents alone is not always effective due to the emergence of drug resistant viral strains and adverse effects associated with antiviral drug treatments [3,4]. Both virus and host-mediated factors contribute to lung pathology in influenza thus, a therapeutic approach targeting both the virus and the host response is desirable [5,6]. Among host factors, cytokine storm and aggravated hyper-inflammatory responses are linked with pulmonary damage and respiratory failure.

Neutrophils are short-lived and terminally differentiated cells of innate immune system. Although excessive neutrophil influx has been well described in pathologic development of acute lung injury, the role of neutrophils in influenza pathogenesis is not completely understood. Tate et al. [7] described beneficial effects of neutrophils by clearing the virus during influenza virus infection in mice. However, several studies have found neutrophilic predominant infiltrations within damaged lungs following several highly pathogenic influenza viral infections [8–10]. Our studies using mouse models have found that excessive neutrophil influx and neutrophil extracellular traps (NETs) contribute to immunopathology in influenza [11–14]. Based on our findings we hypothesize that targeting neutrophil-mediated acute lung injury together with viral growth could be a potential therapeutic strategy in combating severe influenza pneumonia.

NETs are released from neutrophils through a cell death mechanism called NETosis and contain DNA fibers decorated with histones and cytosolic proteins. Persistent accumulations of neutrophils and NETs exacerbate alveolar-capillary damage leading to vascular leak and edema that ultimately results in respiratory failure in mice challenged with a lethal influenza virus [11]. Extracellular traps occlude small airways and blood vessels causing epithelial and endothelial damage within the infected areas of the lung [11]. Further, our initial findings using a mouse lethal influenza infection model demonstrated two possible ways controlling neutrophils/NETs mediated-injury in lethal influenza infection. Extracellular histones released during influenza, are functionally active, induce cytotoxicity in alveolar epithelial and endothelial cells evaluated by lactate dehydrogenase release assay; promote microvascular thrombosis in pulmonary vasculature *in vivo*. Blocking of extracellular histones with anti-histone antibodies significantly decreases lung pathology. Use of a combination of anti-histone antibodies together with an anti-viral agent provides significant protection in lethal influenza challenged mice. We also found various chemokine receptors expressions on neutrophils after infection. Blocking neutrophil recruitment using a chemokine receptor antagonists in combination with an anti-viral agent significantly reduce lung pathology, virus titer and improves survival after lethal influenza challenge in mice. Further studies are in progress testing the combination therapy in a swine-influenza infection setting.
